# Emergency response and the need for collective competence in epidemiological teams

**DOI:** 10.2471/BLT.20.276998

**Published:** 2021-03-02

**Authors:** Amy Elizabeth Parry, Martyn D Kirk, David N Durrheim, Babatunde Olowokure, Samantha Colquhoun, Tambri Housen

**Affiliations:** a National Centre for Epidemiology and Population Health, Research School of Population Health, Australian National University, Building 62, Mills Road, Acton, Canberra, Australian National Territory, 2601 Australia.; b School of Medicine and Public Health, University of Newcastle, New South Wales, Australia.; c Health Emergencies Programme, World Health Organization, Geneva, Switzerland.

## Abstract

**Objective:**

To determine the challenges met by, and needs of, the epidemiology emergency response workforce, with the aim of informing the development of a larger survey, by conducting key informant interviews of public health experts.

**Methods:**

We defined our study population as public health experts with experience of epidemiology deployment. Using purposive sampling techniques, we applied random number sampling to shortlists of potential interviewees provided by key organizations to obtain 10 study participants; we identified three additional interviewees through snowballing. The same interviewer conducted all key informant interviews during May–August 2019. We thematically analysed de-identified transcripts using a qualitative data analysis computer software package.

**Findings:**

Despite our interviewees having a wide range of organizational and field experience, common themes emerged. Interviewees reported a lack of clarity in the definition of an emergency response epidemiologist; the need for a broader range of skills; and inadequate leadership and mentoring in the field. Interviewees identified the lack of interpersonal skills (e.g. communication) and a lack of career progression options as limitations to the effectiveness of emergency response.

**Conclusion:**

The epidemiology emergency response workforce is currently not achieving collective competence. The lack of a clear definition of the role must be addressed, and leadership is required to develop teams in which complementary skills are harmonized and those less experienced can be mentored. Epidemiology bodies must consider individual professional accreditation to ensure that the required skills are being achieved, as well as enabling continual professional development.

## Résumé

**Objectif:**

Identifier les défis et besoins des équipes épidémiologiques chargées de l'intervention d'urgence afin d'étayer l'élaboration d'une enquête plus vaste, en menant des entretiens auprès d'intervenants clés parmi les experts en santé publique.

**Méthodes:**

La population que nous avons définie pour notre étude est composée d'experts en santé publique possédant de l'expérience dans le déploiement en situation épidémique. Nous avons utilisé des techniques d'échantillonnage raisonné pour appliquer un nombre aléatoire aux listes d'intervenants potentiels fournies par les organisations clés, et obtenu ainsi 10 participants. Nous avons sélectionné trois intervenants supplémentaires par effet boule de neige. Les entretiens avec tous les intervenants ont été menés par une seule et même personne entre mai et août 2019. Nous avons examiné les transcriptions anonymisées de façon thématique à l'aide d'un logiciel d'analyse des données qualitatives.

**Résultats:**

Bien que nos intervenants affichent un large éventail d'expériences, tant au sein des organisations que sur le terrain, des thèmes communs sont apparus. Plusieurs d'entre eux ont signalé un manque de clarté dans la définition de l'épidémiologiste chargé de l'intervention d'urgence; ils ont exprimé le besoin de combiner davantage de compétences; ils estiment également que le leadership et l'encadrement sur le terrain sont inadéquats. Par ailleurs, ils ont invoqué l'absence de compétences relationnelles (notamment en matière de communication) et de perspectives d'évolution de carrière comme motifs limitant l'efficacité de l'intervention d'urgence.

**Conclusion:**

À l'heure actuelle, les équipes épidémiologiques chargées de l'intervention d'urgence manquent de compétences collectives. Il faut mieux définir le rôle à jouer et établir un leadership afin de constituer des équipes qui réunissent des compétences complémentaires, où les moins expérimentés peuvent être encadrés. Les autorités épidémiologiques doivent envisager l'octroi d'accréditations pour s'assurer que chacun possède les aptitudes requises, et favoriser le développement professionnel continu.

## Resumen

**Objetivo:**

Identificar los retos y necesidades de los equipos de respuesta a emergencias epidemiológicas para apoyar el desarrollo de una encuesta más amplia, a través de entrevistas con las principales partes interesadas entre los expertos en salud pública.

**Métodos:**

La población que hemos definido para nuestro estudio está compuesta por expertos en salud pública con experiencia en el despliegue de epidemias. Utilizamos técnicas de muestreo intencional para aplicar un número aleatorio a las listas de posibles interesados proporcionadas por las organizaciones clave, lo que dio como resultado 10 participantes. Se seleccionaron otras tres partes interesadas mediante un efecto de bola de nieve. Las entrevistas con todas las partes interesadas fueron realizadas por una persona entre mayo y agosto de 2019. Se revisaron las transcripciones anonimizadas temáticamente utilizando un software de análisis de datos cualitativos.

**Resultados:**

A pesar de que los entrevistados tenían una amplia experiencia, tanto dentro de las organizaciones como sobre el terreno, surgieron varios temas comunes. Varios señalaron la falta de claridad en la definición de un epidemiólogo de respuesta a emergencias, la necesidad de más combinaciones de habilidades y un liderazgo y orientación inadecuados en el campo. Además, citaron la falta de habilidades interpersonales (incluidas las de comunicación) y de oportunidades de desarrollo profesional como razones que limitan la eficacia de la respuesta a las emergencias.

**Conclusión:**

Actualmente, el personal de respuesta a emergencias epidemiológicas no está logrando una competencia colectiva. Hay que abordar la falta de una definición clara de la función, y se necesita liderazgo para desarrollar equipos en los que se armonicen las habilidades complementarias y se pueda tutelar a los menos experimentados. Los organismos de epidemiología deben considerar la acreditación profesional individual para garantizar que se alcanzan las competencias requeridas, además de permitir el desarrollo profesional continuo.

## ملخص

الغرض تحديد الصعوبات التي يواجهها فريق العمل الخاص بالاستجابة لحالات الطوارئ الوبائية واحتياجاته، وذلك بهدف الاعتماد عليه لوضع استطلاع آراء أكبر عن طريق إجراء مقابلات شخصية مع المبلغين الرئيسيين من خبراء الصحة العامة.

الطريقة حددنا فئات المشاركين في الدراسة من خبراء الصحة العامة الذين لديهم خبرة في تفشي الأوبئة. باستخدام آليات هادفة لتحديد العينات، أخذنا عينات بأعداد عشوائية من الأشخاص المحتملين لإجراء مقابلات شخصية معهم والذين اختارتهم منظمات رئيسية من أجل الحصول على 10 مشاركين في الدراسة، وحددنا ثلاثة أشخاص إضافيين لإجراء مقابلات معهم من خلال الانتقاء العشوائي. أجرى مسؤول مقابلات واحد كل المقابلات الشخصية مع المبلغين الرئيسيين خلال الفترة من مايو/أيار إلى أغسطس/آب 2019. أجرينا تحليلاً موضوعيًا للنصوص اللاغية باستخدام برنامج كمبيوتر لتحليل البيانات النوعية.

النتائج على الرغم من أن من تم إجراء المقابلات الشخصية معهم يتمتعون بخبرات واسعة متنوعة مؤسسيًا وميدانيًا، ظهرت أفكار مشتركة. أبلغ من تم إجراء المقابلات الشخصية معهم بعدم الوضوح في تعريف أخصائي الأوبئة المستجيب لحالة الطوارئ؛ وغياب القيادة والتوجيه الكافيين ميدانيًا. أشار من تم إجراء المقابلات الشخصية معهم إلى غياب المهارات الشخصية (مثل التواصل) وغياب خيارات التطور المهني ضمن القيود على فعالية الاستجابة للطوارئ.

الاستنتاج فريق العمل الخاص بالاستجابة لحالات الطوارئ الوبائية لا يحقق حاليًا كفاءة جماعية. يجب التعامل مع غياب التعريف الواضح للدور، وهناك حاجة لأن تتولى قيادة إنشاء فرق تتجانس فيها المهارات التكميلية ويمكن فيها الإشراف على من لديهم قدر أقل من الخبرة. يجب أن تضع الجهات الخاصة بالتعامل مع الأوبئة في اعتبارها الاعتماد المهني للأفراد لضمان تحقيق المهارات المطلوبة إلى جانب تمكين التطور المهني المتواصل.

## 摘要

**目的:**

旨在确定流行病学应急响应人员所面临的挑战及其需求，从而通过对公共卫生专家展开关键人物访谈，了解大型调查的进展。

**方法:**

我们规定，调查人员是指具备流行病学调查部署经验的公共卫生专家。我们使用立意抽样技术，通过对主要组织提供的潜在受访者名单进行随机抽样，确定了 10 名调查参与者；我们还通过滚雪球方式确定了另外三名受访者。在 2019 年 5 月至 8 月期间由同一位访谈人员对所有受访者展开关键人物访谈。我们利用定性数据分析计算机软件，对不包含识别信息的记录进行了专题分析。

**结果:**

尽管我们的受访者拥有各种不同的组织和现场经验，但他们提出了相同的问题。受访者报告时称，应急响应流行病学家的定义不明确；需要范围更广的一系列技能；以及现场领导和指导能力不足。受访者认为人际交往能力（例如沟通）不足和未提供职业发展选择机会会限制应急响应的有效性。

**结论:**

流行病学应急响应人员目前尚不具备集体实践能力。必须解决角色定义不明确的问题，且需要确定带领团队发展的领导人，从而完美实现团队成员技能互补，并对经验不足的人进行指导。流行病学机构必须考虑个人专业资格，以确保其具备所需的技能，并能够实现持续职业发展。

## Резюме

**Цель:**

Определить проблемы, с которыми сталкиваются работники служб реагирования на чрезвычайные эпидемиологические ситуации, и их потребности с целью информированной разработки более масштабного анкетирования посредством собеседований с ключевыми информантами из числа специалистов в области общественного здравоохранения.

**Методы:**

Авторы определили в качестве популяции исследования специалистов в области общественного здравоохранения с опытом работы в эпидемиологических очагах. Используя методики преднамеренной выборки, авторы случайным образом выбирали то или иное количество участников из прошедших предварительный отбор списков потенциальных интервьюируемых лиц, предоставленных ключевыми организациями, чтобы набрать 10 участников (через интервьюируемых лиц нами были обнаружены 3 дополнительных кандидата). На протяжении мая-августа 2019 года один и тот же интервьюер опрашивал всех ключевых информантов. Деидентифицированные расшифровки собеседований были тематически проанализированы при помощи компьютерной программы для количественного анализа данных.

**Результаты:**

Несмотря на то что интервьюируемые лица представляли самые разные организации и обладали весьма разнообразным практическим опытом работы, мы смогли выделить некоторые общие моменты. Интервьюируемые лица говорили о том, что роль эпидемиолога, работающего в условиях ликвидации чрезвычайной ситуации, определена недостаточно четко, что у такого специалиста должен быть более широкий диапазон навыков, и о том, что в полевых условиях осуществляется недостаточное по качеству руководство и наставничество. По мнению интервьюируемых лиц, основными препятствиями в деле эффективной ликвидации чрезвычайных ситуаций является недостаток навыков межличностного контакта (общения) и нехватка возможностей карьерного роста.

**Вывод:**

В настоящее время работники служб реагирования на чрезвычайные эпидемиологические ситуации не достигают коллективной компетенции. Следует обратить внимание на недостаточно четкое определение роли специалиста, а руководителям следует позаботиться о создании команд, в которых взаимодополняющие навыки участников гармонично объединены, а недостаточно опытные коллеги могут получить помощь наставников. Эпидемиологическим органам следует задуматься о необходимости индивидуальной профессиональной аккредитации, которая гарантировала бы наличие у кандидатов требуемых навыков, а также о создании возможностей для непрерывного профессионального роста.

## Introduction

Global public health emergencies are becoming increasingly frequent and complex, partly driven by fragile and underresourced health-care systems. For an effective response to such emergencies, investment in a strong and equitable health-care system and workforce, to ensure the necessary combination of competencies, knowledge and skills, is essential.^1^^–^^3^ Signatory States to the International Health Regulations (IHR) 2005 are required to have an agreed minimum capacity for infectious disease surveillance, alert and response.^4^ However, IHR evaluations have shown that these minimum standards are rarely met;^2^ the ongoing response to the coronavirus disease 2019 (COVID-19) pandemic provides further confirmation of the discordance between reported capacity and response.^5^

Applied epidemiologists work to prevent excess mortality and morbidity by ensuring that appropriate investigation and control activities are implemented in an effective and timely manner, and provide information for evidence-based decision-making.^6^^–^^9^ A commonly agreed core competency of an applied epidemiologist is an understanding of epidemiological methods for outbreak response and public health surveillance.^10^ However, despite being key members of the health emergency workforce, the definition of epidemiology training can vary widely to include: an epidemiology course-work unit as part of a Master of Public Health degree; a doctorate degree on a specific epidemiology research topic; or even specialized training in field epidemiology such as the internationally recognized field epidemiology training programmes. Participation in such a programme does not confer a certain range of knowledge either, as there are three levels: frontline (≤ 6 months), intermediate (9 months–1 year) and advanced (2 years).^9^^,^^11^ There is no professional accreditation for epidemiologists, and no recognition of sub-specializations such as emergency response. 

**Fig. 1:**
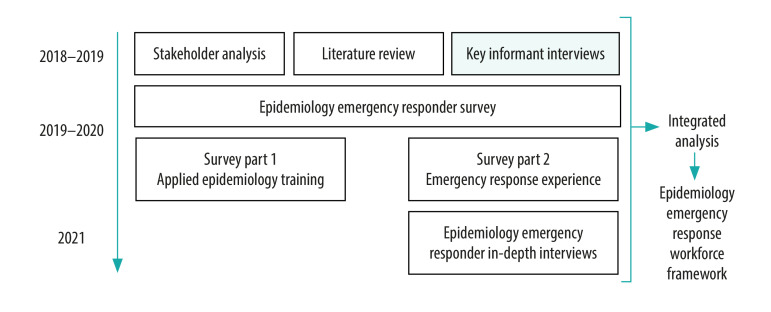
How the key informant interviews form part of a larger epidemiology emergency response workforce study, 2019–2020

Note: The key informant interview module is highlighted in light blue.

To inform the development of an epidemiology emergency responder survey,^12^^,^^13^ alongside stakeholder analysis and an unpublished literature review, we conducted key informant interviews (Fig. 1) to investigate the challenges met by, and the needs of, the epidemiology emergency response workforce. We describe and discuss the major themes important to the interviewed public health experts, as revealed during these key informant interviews.

## Methods

### Study population

We defined our study population as individuals with experience of public health emergencies. Specifically, we wanted to interview people with direct experience of epidemiology deployment and of supervising epidemiologists in the field, or who had supported the deployment of epidemiologists. Interviewees were limited to those who could communicate in English for the interview.

We used a combination of purposive sampling techniques to identify key informant interviewees.^14^^,^^15^ We asked key organizations who deploy epidemiologists to emergencies to identify a shortlist of people for interview based on the above criteria. To ensure proportional representation from a range of global agencies, we set minimum quotas for each organization based on their frequency of deployment of epidemiologists. Of an anticipated sample of 10 interviews, we aimed to interview four individuals employed by the World Health Organization (WHO), and two individuals employed by each of the Global Outbreak Alert and Response Network, Médecins Sans Frontières and the United States Centers for Disease Control and Prevention. From the shortlists provided by these four organizations, we selected interviewees by random number sampling using Stata version 15 (StataCorp, College Station, United States of America, USA). 

We identified the remaining interviewees via a snowballing approach, where we asked each of the 10 interviewees obtained as above to name potential additional participants. To reduce the clustering of networks, we invited a maximum of two candidates to participate in our study per interviewee from the list compiled through snowballing.

### Data collection

We selected our interview questions according to issues raised in our literature review and in open interviews conducted during an initial scoping exercise in 2018. For the latter, we used a convenience sample of three epidemiologists who had worked during emergencies in resource-constrained settings. We aimed to acquire practical details on epidemiologist deployment, as well as all interviewees’ opinions on roles and required skills for applied epidemiology fieldwork, performance management in the field and workforce upskilling during deployments.

We conducted our key informant interviews during May–August 2019. We provided all participants with a study information sheet that outlined the project and expectations in plain language. Participation was voluntary, and each interviewee provided written consent. The same interviewer conducted all semi-structured key informant interviews either by telephone or internet communications, and continued the interviews until saturation (the point at which no new information, themes or issues were being discussed).^16^^,^^17^

The interviewer asked questions in both an iterative style to ensure flow and a probing style to obtain clear answers.^18^ We recorded all interviews in full and then used auto-transcription software Sonix (sonix.ai, San Francisco, USA) to transcribe these verbatim. The principal investigator then verified the accuracy of the interview transcriptions with the actual recordings. We provided participants with the opportunity to request a copy of their transcripted interview, and to comment if it was felt necessary (e.g. if they felt their anonymity was compromised).

### Analysis

We redacted identifying information from the transcripts, then conducted data familiarization through listening to the recordings and reading the interview transcripts.^19^ The transcripts were coded and then thematically analysed using the qualitative data analysis software NVivo 11 (QSR International Pty Ltd, Melbourne, Australia). We defined a theme as a recognized pattern within the data.^20^^,^^21^ We conducted thematic analysis iteratively, and used inductive coding to identify relationships within the data without using a pre-existing frame.^16^^,^^20^ We then reviewed and summarized codes before interpreting them for meaning.^20^ To ensure that we considered the direct as well as underlying ideas that were discussed, we conducted both semantic and latent analysis of the interviews.^20^

We adopted a pragmatic interpretivist approach^22^^–^^24^ and aimed to understand the interview findings through a process of interpretation, as viewed through our personal culture and experience.^23^ Our findings are an interpretation of the lived experiences of the participants, bound by both time and context.^23^ Because the principal investigator of this study has worked in both acute and protracted emergencies as an epidemiologist, findings are also interpreted through her lived experience. 

### Ethics

The Australian National University Human Research Ethics Committee provided approval (identification no. 2019–521) of our study.

## Results

### Interviewee demographics

We had to invite a total of 36 senior public health experts to participate in our study, identified via repeated random number sampling, before obtaining the agreement of 10 to be interviewed. The other three participants were obtained via snowballing. Study participants reported deployment to the largest health emergencies of the past decade, including: outbreaks of avian influenza, cholera, Ebola virus disease, Lassa fever, measles and severe acute respiratory syndrome; the aftermaths of natural disasters (e.g. Cyclone Winston in Fiji in 2016 and Cyclone Idai in Mozambique in 2019); and the refugee crises at Cox’s Bazar (Bangladesh) and Kharaz (Yemen). Interviewees were mostly male (61.5%; 8/13) and most were employed by WHO at the time of interview (46.2%, 6/13). Other interviewees worked at Médecins Sans Frontières (3/13, 23.1%), the Global Outbreak Alert and Response Network (3/13, 23.1%) or an Australian health department (1/13, 7.7%) at the time of interview. We encouraged all interviewees to discuss previous experience in other public health agencies before their current employment, including the International Federation of Red Cross and Red Crescent Societies, Public Health England, the Robert Koch Institute, the United States Centers for Disease Control and Prevention, the United Nations Food and Agriculture Organization and WHO (including headquarters and both regional and country offices). 

### Interview themes

Although the interviewees reported a diverse range of organizational and field experience, three common themes emerged. Interviewees reported (i) a lack of clarity in defining the role of an epidemiologist during emergency responses; (ii) the need for a broader range of skills over and above traditional epidemiology skills; and (iii) inadequate leadership and mentoring in the field. 

#### (i) Role

In commencing with the simple question: “Who are emergency response epidemiologists and what do they do?”, we uncovered a key issue of role classification (Box 1). Interviewees stated that the title epidemiologist was largely self-nominated, and reported that the emergency response workforce was largely individualistic with a high turnover and a widely varying combination of skills and experience.

Box 1 **Interviewee quotes on the role of epidemiologists from study to determine epidemiology workforce needs during emergency response, 2019**

“The job title ‘epidemiologist’ is not specific to the skill set that people bring, so you’ll find a huge range of people doing that job.”

“There are all sorts of tasks that you might be asked to do within one job description.”

“When you’re in the field you’ve got to find the work and create your own work because often people know your job description is a bit loose.”

We also learnt that epidemiology responders are often invited to join teams based on their availability, rather than their specialist role or any particular expertise required within the team. Interviewees reported how, when deployed to an emergency, they often had no or nonspecific terms of reference and had to begin by determining how their expertise could support the response (Box 1). With experience, epidemiology responders could understand their role and how they contributed to the response, and were able to judge what the priorities were. However, with no clear terms of reference or guidance, key informants reported how they had observed less-experienced responders focus on what they knew or felt comfortable doing, rather than response priorities.

We ascertained differing views regarding whether specific terms of reference were needed. Needs and priorities change rapidly during emergencies, and the role of the epidemiologist is to adapt to what is needed at the time. Interviewees indicated that having clearer roles and skill definitions would improve performance, especially among less-experienced emergency response epidemiologists.

#### (ii) Required skills

When discussing skills required by emergency response epidemiologists, interviewed experts overwhelmingly focused on skills that were not traditionally the focus of epidemiology training programmes. Interpersonal skills, particularly communication, listening and the ability to work effectively with a range of people and teams, were reported as being extremely important (Box 2 and Box 3). The interviewees did discuss the need for technical epidemiology skills, such as data collection and analysis; however, interviewees frequently stated that high-level epidemiology skills were not required for emergency response. Instead, what was generally considered to be of higher value was the ability to apply basic epidemiology skills to a range of different health emergencies. Participants reported that the application of technical skills was linked to experience, as well as a clear understanding of their role within a multidisciplinary team and the overall response (Box 3).

Box 2 **Skills needed according to interviewees in study to determine epidemiology workforce needs during emergency response, 2019**

Interpersonal: adaptability, communication, creativity, open-mindedness, humility, team-working

Management: communication, conflict resolution, coordination, leadership, observation, prioritization, team building

Technical: epidemiology knowledge

Box 3 **Interviewee quotes on the need for social and communication skills from study to determine epidemiology workforce needs during emergency response, 2019**

**Communication**

“If things don’t go well it’s less often due to the lack of technical skills and more often due to the lack of ability to communicate effectively and set priorities among the stakeholders.”

“There needs to be more space for listening.”

**Collaboration**

“Most missions don’t fail because of technical incompetence, they fail because of…the softer skills: the teamwork, the coordination.”

“I think a lot of people [are] working very strong in their silo and not looking left and right.”

“Some people might be technically good, but they lack field engagement.”

“Be flexible, open minded, not have a runaway ego. You know, a lot of those things are true for all people responding in those events… difficult to train for.”

We observed that communication was the skill most commonly discussed. Interviewees considered that communication skills were underrecognized, but critical for success (Box 3). Strong communication skills and the ability to work in cross-cultural multidisciplinary teams were reported as the core competencies required of emergency response epidemiologists (Box 3). The discussion around communication covered many areas including listening, cross-cultural communication, communication within and between teams and organizations, and interview skills to obtain required information. Participants reported that the ability to communicate technical findings to local government, responders and communities was also crucial. Interviewees were concerned that, although communication skills are vital, they are highly linked to personality and difficult to teach and learn. Our study participants stated that finding people with both the preferred experience and these critical communication skills was difficult.

The ability to build relationships and to coordinate with peers were also identified as important skills that are not easily taught, but are learnt through experience (Box 3). Interviewees reported that a common challenge was colleagues who worked in a silo (i.e. preferred to work alone) rather than connecting with team members or other agencies. Respondents acknowledged that finding the required combination of skills (Box 2) in one person was rare, and that there was a need to assemble teams in which members could provide complementary skills.

#### (iii) Leadership and mentoring

We noted that the need for leadership and mentoring during an emergency response was a consistent theme emerging from the interviews. When asked what an epidemiologist needed to respond effectively during an emergency, most interviewees replied “experience”. However, interviewees also identified the paradox of this statement: experienced people were not always available, and less-experienced epidemiologists were more readily available but needed guidance. Interviewees acknowledged that an emergency response was not an ideal training environment for someone without experience, but recognized that mentoring and supervisory mechanisms to support less-experienced responders could be a solution (Box 4).

Box 4 **Interviewee quotes on the need for leadership and mentoring from study to determine epidemiology workforce needs during emergency response, 2019**

**Leadership**

“Depends on the…team lead on whether or not you do something meaningful as the epi[demiology] team.”

**Mentoring**

“It’s a matter of supporting them so they can be successful.”

“It can be a very stressful situation for the epi[demiologist] in the field if you really have no clue what to do.”

During the interviews we explored how to manage inexperience and provide mentoring during an emergency response. It was recognized that experience is not a skill and cannot be taught in the traditional teaching environment. Interviewees discussed the value of field epidemiology training programmes, which provide hands-on experience during learning; however, not all trainees are exposed to emergency response scenarios. Interviewees discussed the importance of ensuring that, when in the field, performance management and mentoring were available to ensure successful deployments (Box 4). However, our study participants reported that this was often hampered by a high staff turnover, short deployment periods and limited technical briefings before and during deployment.

We also discussed career paths for emergency response epidemiologists during the interviews. Study participants were concerned that the limited options for career progression within emergency response hampered the effectiveness of any response, as well as the opportunity to gain experience and skills as a mentor or team leader. Interviewees described how they had encountered responders who, with limited experience in the field, were tasked with leadership roles that they often were not trained for or supported in.

## Discussion

The epidemiology workforce is an important component of emergency response; however, our key informant interviews highlight multiple issues being faced by the epidemiology workforce, limiting the effectiveness of emergency response. An underlying theme in our interviews was the need for emergency response teams that draw on collective competence rather than individual ability.^25^ Our interviews revealed that individuals often worked independently, and therefore ineffectively, during emergency response.

Collective understanding of roles within and between teams is important for an effective response.^1^^,^^26^ Our findings indicate that, despite the clarity of specific tasks (e.g. the epidemiologist’s role during rapid needs assessments or as a member of a rapid response team), the overarching role of an epidemiologist throughout the response is not clearly or consistently understood. Clarifying the latter could lead to greater utilization of core skills and competencies, and greater efficiency in the use of often-scarce skilled emergency response human resources. Our results conform with the need to develop competencies and certification for health professionals who respond to public health emergencies, as already identified in reviews of the 2013–2016 Ebola virus disease outbreak response in West Africa.^1^^,^^27^ The lack of professional accreditation leads to inconsistencies in competencies, knowledge and skills, with a resulting widespread misunderstanding of what an emergency response epidemiologist is and can do. Without clear guidance, the epidemiologist’s role has been interpreted differently by health organizations and emergency response teams. This ambiguity creates confusion in the field, and can contribute to reduced effectiveness in team response.

The knowledge and skill requirements of the epidemiology workforce also need to be better clarified and understood. Our study findings identified that epidemiologists, especially those who respond to emergencies, require a wide breadth of skills beyond the core methodological and technical epidemiology understanding. Research has shown that, when responders do not have the necessary technical, cultural or communication skills, there is a risk of causing more harm than good.^28^^,^^29^ Strong communication skills and the ability to work in cross-cultural multidisciplinary teams are core competencies required of emergency response epidemiologists; however, analysis of our interviews show that these skills are repeatedly identified as lacking.^26^^,^^30^

Within public health emergency responses, multiple teams with a wide range of technical skills need to work together to ensure appropriate action and control measures are being implemented.^31^ Interdependency among and within teams is therefore essential to achieve collective competence.^25^ Our interviewees discussed relationship building and coordination as important skills that are not easily taught, but are learnt through experience. Interviewees reported that a common problem was responders focusing solely on their own task and results, rather than connecting with team members or other agencies. Team interdependency is also associated with leadership, mentoring and performance management – all themes that emerged from our interviews. Interviewees discussed how public health emergency response requires strong and clear leadership. Again, this confirms what is already known; a study of Ebola response epidemiologists in West Africa,^26^ student reviews of field epidemiology training^8^^,^^32^^,^^33^ and a study of epidemiology graduates in the USA all reported that leadership was a commonly encountered gap in training.^34^

Our study had several limitations. The interviews conducted during our research aimed to understand processes and experiences, not to analyse the distribution of these experiences.^16^ Although we aimed to minimize the impact of bias, there exists a limited number of people with the knowledge and experience required to participate in the key informant interviews. Organizational shortlisting of possible candidates may have led to the selection of candidates with certain organization-approved views on emergency response. Snowballing may also have introduced bias, as participants identified in that way were from the same network as our other interviewees and may have shared certain opinions.^35^ The principal investigator of this study has epidemiology emergency response experience, which may have influenced the questions asked and the information obtained. However, our selection criteria of possible participants, multistep sampling method and minimizing of clustering all aimed to reduce bias as far as possible. Further, our study benefited from the semi-structured nature of our interviews, enabling interviewees to focus on what they believed to be important.

Our findings indicate that the epidemiology emergency response workforce is currently not achieving collective competence.^25^ Addressing the lack of a clear definition of the emergency response epidemiologist requires a focus on strengthening the interdependency within teams. Leadership is required to develop teams in which traditional and non-traditional epidemiology skills are harmonized, and in which those with experience are matched with those who would benefit from a mentor. Epidemiology bodies must consider individual professional accreditation to ensure that the required skills for generalist and subspecialty epidemiologists are being achieved, as well as enabling continual professional development. As the COVID-19 pandemic continues, teams need support to optimize the functioning of critical staff.^36^

## References

[1] Checchi F, Waldman RJ, Roberts LF, Ager A, Asgary R, Benner MT, et al. World Health Organization and emergency health: if not now, when? BMJ. 2016 1 28;352:i469. 10.1136/bmj.i469 26821569

[2] Global Preparedness Monitoring Board. A world at risk: annual report on global preparedness for health emergencies [internet]. Geneva: World Health Organization; 2019. Available from: https://apps.who.int/gpmb/assets/annual_report/GPMB_annualreport_2019.pdf [cited 2021 Feb 7].

[3] Kruk ME, Myers M, Varpilah ST, Dahn BT. What is a resilient health system? Lessons from Ebola. Lancet. 2015 5 9;385(9980):1910–12. 10.1016/S0140-6736(15)60755-3 25987159

[4] International Health Regulations (2005). Geneva: World Health Organization; 2005. Available from: https://www.who.int/ihr/publications/9789241580496/en/ [cited 2021 Feb 7].

[5] The global health security index [internet]. Washington, DC, Baltimore and London: Nuclear Threat Initiative, Johns Hopkins Center for Health Security and Economist Intelligence Unit; 2021. Available from: https://www.ghsindex.org/ [cited 2021 Feb 7].

[6] Connolly MA, Gayer M, Ryan MJ, Salama P, Spiegel P, Heymann DL. Communicable diseases in complex emergencies: impact and challenges. Lancet. 2004 11 27;364(9449):1974–83. 10.1016/S0140-6736(04)17481-3 15567014

[7] O’Carroll P, Kirk M, Baggett K, Herrera D. The global field epidemiology roadmap. Decatur: The Task Force for Global Health; 2018. Available from: https://www.tephinet.org/sites/tephinet/files/content/attachment/2018-11-26/The%20Global%20Field%20Epidemiology%20Roadmap_11.26.18.FINAL_.pdf [cited 2021 Feb 7].

[8] Nsubuga P, White M, Fontaine R, Simone P. Training programmes for field epidemiology. Lancet. 2008 2 23;371(9613):630–1. 10.1016/S0140-6736(08)60281-0 18295009

[9] Bensyl DM, King ME, Greiner A. Applied epidemiology training needs for the modern epidemiologist. Am J Epidemiol. 2019 5 1;188(5):830–5. 10.1093/aje/kwz052 30877297 PMC6608580

[10] Traicoff DA, Walke HT, Jones DS, Gogstad EK, Imtiaz R, White ME. Replicating success: developing a standard FETP curriculum. Public Health Rep. 2008;123(Suppl 1:28–34. 10.1177/00333549081230S109 18497016 PMC2233740

[11] Training programs. Decatur: Training Programs in Epidemiology and Public Health Interventions Network; 2020. Available from: https://www.tephinet.org/training-programs [cited 2021 Feb 7].

[12] Parry AE, Kirk MD, Durrheim DN, Olowokure B, Housen T. Study protocol: building an evidence base for epidemiology emergency response, a mixed-methods study. BMJ Open. 2020 6 29;10(6):e037326. 10.1136/bmjopen-2020-037326 32601115 PMC7328751

[13] Parry AE, Kirk MD, Durrheim DN, Olowokure B, Housen T. Study protocol: building an evidence base for epidemiology emergency response, a mixed-methods study. BMJ Open. 2020 6 29;10(6):e037326. 10.1136/bmjopen-2020-037326 32601115 PMC7328751

[14] Padget DK. Qualitative and mixed methods in public health. Newbury Park: SAGE Publications; 2012.

[15] Teddlie C, Yu F. Mixed methods sampling: a typology with examples. J Mixed Methods Res. 2007 1;1(1):77–100. 10.1177/1558689806292430

[16] Liamputtong P. Qualitative research methods. South Melbourne: Oxford University Press Australia; 2009.

[17] Bowen GA. Naturalistic inquiry and the saturation concept: a research note. Qual Res. 2008 2 1;8(1):137–52. 10.1177/1468794107085301

[18] Guest G, MacQueen KM, Namey EE. Chapter 2: Planning and preparing the analysis. Applied thematic analysis. Newbury Park: SAGE Publications; 2012. 10.4135/9781483384436.n2

[19] Clarke V, Braun B. Teaching thematic analysis: overcoming challenges and developing strategies for effective learning [data repository]. Bristol: UWE Bristol Research Repository; 2013. Available from: https://uwe-repository.worktribe.com/preview/937606/Teaching%20thematic%20analysis%20Research%20Repository%20version.pdf [cited 2021 Feb 7].

[20] Braun V, Clarke V. Using thematic analysis in psychology. Qual Res Psychol. 2006 1 1;3(2):77–101. 10.1191/1478088706qp063oa

[21] Joffe H. Chapter 15: Thematic analysis. In: Harper D, Thompson A, editors. Qualitative research methods in mental health and psychotherapy: a guide for students and practitioners. Chichester: Wiley; 2012. pp. 209–23.

[22] Cameron R. Mixed methods research: the five Ps framework. Electron J Bus Res Methods. 2011;9(2):96–108.

[23] Grbich C. Qualitative data analysis: an introduction. 2nd ed. London: SAGE Publishing; 2012.

[24] Johnson RB, Onwuegbuzie AJ. Mixed methods research: a research paradigm whose time has come. Educ Res. 2004;33(7):14–26. 10.3102/0013189X033007014

[25] Boreham N. A theory of collective competence: challenging the neo-liberal individualisation of performance at work. Br J Educ Stud. 2004 3 1;52(1):5–17. 10.1111/j.1467-8527.2004.00251.x

[26] Holding M, Ihekweazu C, Stuart JM, Oliver I. Learning from the epidemiological response to the 2014/15 Ebola virus disease outbreak. J Epidemiol Glob Health. 2019 9;9(3):169–75. 10.2991/jegh.k.190808.002 31529934 PMC7310819

[27] Gostin LO, Friedman EA. Ebola: a crisis in global health leadership. Lancet. 2014 10 11;384(9951):1323–5. 10.1016/S0140-6736(14)61791-8 25306563

[28] Hunt MR. Moral experience of Canadian healthcare professionals in humanitarian work. Prehosp Disaster Med. 2009 Nov-Dec;24(6):518–24. 10.1017/S1049023X00007445 20301070

[29] Chiu C-Y, Lonner WJ, Matsumoto D, Ward C. Cross-cultural competence: theory, research, and application. J Cross Cult Psychol. 2013;44(6):843–8. 10.1177/0022022113493716

[30] Forbes O, Davis S, Dyda A, Rosewell A, Williams S, Moffatt C, et al. Expert perspectives on outbreak investigation training: a quality improvement exercise. Glob Biosecurity. 2020 7 7;1(4):32. 10.31646/gbio.53

[31] Emergency Response Framework (ERF) 2nd edition. Geneva: World Health Organization; 2017. Available from: https://www.who.int/hac/about/erf/en/ [cited 2021 Feb 7].

[32] Schneider D, Evering-Watley M, Walke H, Bloland PB. Training the global public health workforce through applied epidemiology training programs: CDC’s experience, 1951–2011. Public Health Rev. 2011 6;33(1):190–203. 10.1007/BF03391627

[33] Jones DS, Dicker RC, Fontaine RE, Boore AL, Omolo JO, Ashgar RJ, et al. Building global epidemiology and response capacity with field epidemiology training programs. Emerg Infect Dis. 2017 12;23(13 Suppl 1:S158–65. 10.3201/eid2313.170509 29155658 PMC5711325

[34] Samet JM, Brownson RC. Epidemiology in a changing world. Am J Prev Med. 2014 11;47(5) Suppl 3:S383–5. 10.1016/j.amepre.2014.07.012 25439261

[35] Onwuegbuzie AJ, Collins KMT. The role of sampling in mixed methods-research: enhancing inference quality. KZfSS Köln Z Für Soziol Sozialpsychologie. 2017 10;69 S2:133–56. 10.1007/s11577-017-0455-0

[36] Yong E. The pandemic experts are not okay. The Atlantic [internet]. 2020 Jul 7. Available from: https://www.theatlantic.com/health/archive/2020/07/pandemic-experts-are-not-okay/613879/ [cited 2021 Feb 7].

