# Corrigendum

**DOI:** 10.2471/BLT.21.100721

**Published:** 2021-07-01

In: Parry AE, Kirk MD, Durrheim DN, Olowokure B, Colquhoun S, et al. Emergency response and the need for collective competence in epidemiological teams. Bull World Health Organ. 2021 May 1;99(5):351–358, 

On page 357, reference 13 should read as follows: 

Parry AE, Kirk MD, Durrheim DN, Olowokure B, Colquhoun S, et al. Shaping applied epidemiology workforce training to strengthen emergency response: a global survey of applied epidemiologists, 2019–2020. Hum Resour Health 19, 58 (2021). https://doi.org/10.1186/s12960-021-00603-1

